# Bioactive Glass-Ceramic Scaffolds Coated with Hyaluronic Acid–Fatty Acid Conjugates: A Feasibility Study †

**DOI:** 10.3390/jfb14010026

**Published:** 2023-01-02

**Authors:** Stefania De Luca, Valentina Verdoliva, Saeid Kargozar, Francesco Baino

**Affiliations:** 1 Institute of Biostructures and Bioimaging, National Research Council, 80134 Naples, Italy; 2 Department of Environmental, Biological and Pharmaceutical Sciences and Technologies, University of Campania “Luigi Vanvitelli”, Via Vivaldi 43, 81100 Caserta, Italy; 3 Tissue Engineering Research Group (TERG), Department of Anatomy and Cell Biology, School of Medicine, Mashhad University of Medical Sciences, Mashhad 917794-8564, Iran; 4 Institute of Materials Physics and Engineering, Department of Applied Science and Technology, Politecnico di Torino, Corso Duca degli Abruzzi 24, 10129 Torino, Italy

**Keywords:** hyaluronic acid, green synthesis, coating, scaffold, bioactive glass, bioactivity, microwave irradiation

## Abstract

Promoting bone healing is a key challenge in our society that can be tackled by developing new implantable biomaterials provided with regenerative properties. In this work, the coating of three-dimensional porous glass-derived scaffolds with hyaluronic acid (HA)-fatty acids was investigated for the first time. The starting scaffolds, based on bioactive silicate glass, were produced by foam replication followed by sintering; then, HA-palmitate and HA-oleate conjugate coatings were deposited on the scaffold struts through a dipping procedure. FT-IR analysis confirmed the successful deposition of the coatings on the surface and struts of the scaffolds, the foam-like architecture of which was maintained as assessed by SEM investigations. The in vitro bioactivity of the HA–fatty-acid-coated scaffolds was studied by immersion tests in simulated body fluid and the subsequent evaluation of hydroxyapatite formation. The deposition of the polymeric coating did not inhibit the apatite-forming ability of scaffolds, as revealed by the formation of nanostructured hydroxyapatite agglomerates 48 h from immersion. These promising results motivate further investigation of these novel bioactive systems, which are expected to combine the bone-bonding properties of the glass with the wound-healing promotion carried out by the polymeric conjugates.

## 1. Introduction

Tissue engineering, along with biomaterial-based medicine, is employed to induce the regeneration process of living tissues and to make it as quick as possible [1]. Specifically, bone-tissue engineering is addressed to repair the injured parts of the skeletal system using synthetic biocompatible materials [2]. In this regard, bioactive glasses are considered ideal materials for bone regeneration owing to their peculiar inherent properties [3]. They are able to improve vascularization, osteoblast adhesion, enzyme activity, and the differentiation of mesenchymal stem cells [4,5]. Bioactive glasses are often manufactured as three-dimensional (3D) porous scaffolds acting as templates to stimulate and support the growth of regenerated tissue [6,7]. In addition, the combination of bioactive glasses with a biodegradable natural polymer deposited on their surface was found to enhance bone formation [8]. In this regard, hyaluronic acid (HA) is widely employed in regenerative treatments [9,10,11,12,13]. In fact, it is a natural polymer found in the human body and is often used to stimulate extracellular matrix microenvironments and cell activities such as attachment and proliferation. Moreover, HA is involved in many biological responses, including bone regeneration, angiogenesis, inflammation, and wound healing [10]. It is noteworthy to underline that native HA shows poor mechanical properties and is degraded by hyaluronidase: thus, HA-based systems with improved and tailored characteristics need to be developed. In this regard, the chemical modification of HA, and its combination with other materials, is useful to improve its strength and bioactivity. HA amphiphilic conjugates were obtained by functionalizing HA with substituents that decrease its aqueous solubility and are able to self-assemble in micellar aggregates [14,15]. Naturally occurring fatty acids are typically employed as hydrophobic moieties to be conjugated to the hydrophilic HA skeleton. The system obtained contains moieties that can be considered natural products since they are usually provided by natural resources. In addition to the HA’s broad spectrum of biological activities, the natural fatty acids (palmitic and oleic acids) are able to kill or halt the growth of several pathogenic bacterial strains. This allows for the hypothesis that bioactive glasses incorporating on their surface HA–fatty-acid conjugates could promote successful treatment, ensuring increased bone tissue repair, healing, and regeneration.

Herein, we report the characterization of a couple of novel systems based on bioactive glasses coated with biocompatible and beneficial HA-based conjugates, alongside their envisaged employment as bone tissue-engineering materials.

## 2. Results and Discussion

### 2.1. HA–Fatty-Acid Conjugates

**Figure 1:**
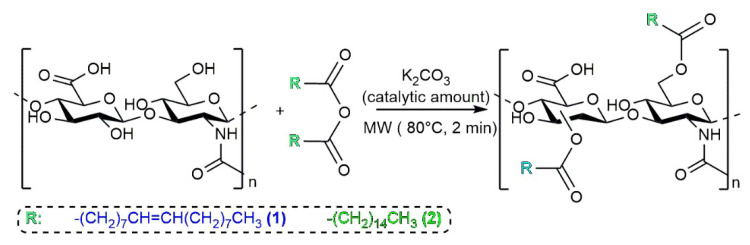
Synthesis strategy of HA–fatty-acid conjugates.

The synthesis protocol to prepare HA–fatty-acid conjugates was reported elsewhere in detail [16]. The esterification reaction of activated fatty acids by the alcoholic functionality of HA was promoted by microwaves without any solvent in order to obtain HA-oleate and HA-palmitate conjugates (Figure 1) [17].

**Figure 2:**
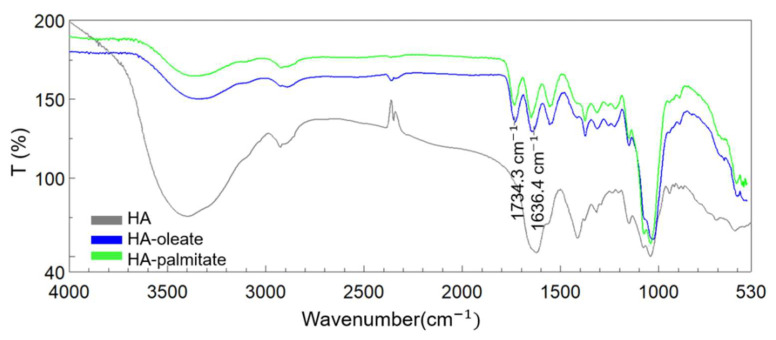
FT-IR spectra of HA alone (grey), HA-palmitate (green), and HA-oleate (blue).

The obtained conjugates were analyzed by FT-IR spectroscopy [18]. The spectra of HA–oleate and HA–palmitate samples are reported in Figure 2; a spectrum of the native HA is also included for comparison purposes. The region of the carbonyl groups takes into account the band of the HA-COO^−^ (~1636 cm^−1^) and the new band that appeared at 1734 cm^−1^, which is attributable to the esterification of the fatty acid (Figure 2) [16].

### 2.2. Coating Procedure of the HA-Based Conjugates on Bioactive Glass Scaffolds

**Figure 3:**
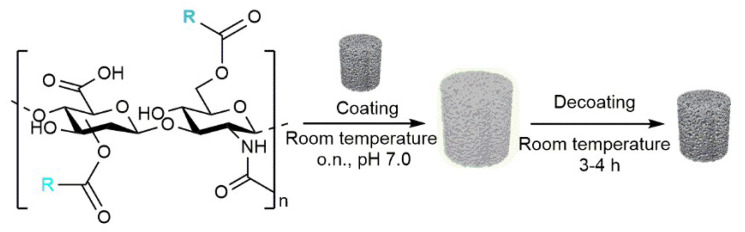
Coating of bioactive glass scaffolds with HA–fatty acids conjugates (center) and decoating (i.e., coating removal) procedure (right).

The obtained conjugates were successively deposited on glass-derived scaffolds to verify a potential effect on their bioactive properties. After the dissolution of each specific HA–fatty acid conjugate in neutral water, pieces of bioactive glass scaffolds were soaked in the obtained solutions. The coating was performed by leaving the mixtures overnight and under gentle shaking at room temperature (Figure 3).

**Figure 4:**
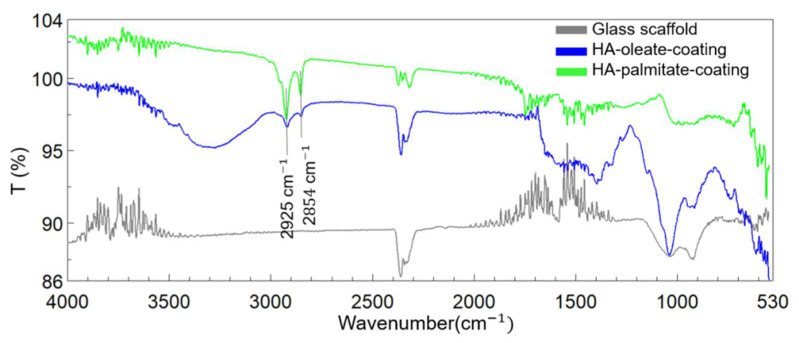
FT-IR spectra of the reference glass scaffold (grey), HA-palmitate-coating (green), and HA-oleate-coating (blue).

Subsequently, the mixtures were lyophilized and the scaffolds were analyzed by FT-IR spectroscopy using the ATR accessory of the instrument. The spectra of HA–oleate- and HA–palmitate-coated glass scaffolds were recorded; a spectrum of the uncoated glass scaffold was also included in the figure for comparison purposes. The region between 750 and 1100 cm^−1^ strongly characterized the glass structure and was related to both Si-O and O-Si-O vibrations. The appearance of new bands at 2940 and 2960 cm^−1^ took into account the fatty acid chain C-H stretching (Figure 4). It is likely that their strong intensity was due to the alkyl chains protruding toward the external glass surface.

### 2.3. Conjugate-Coated Bioactive Glass Scaffolds

**Figure 5:**
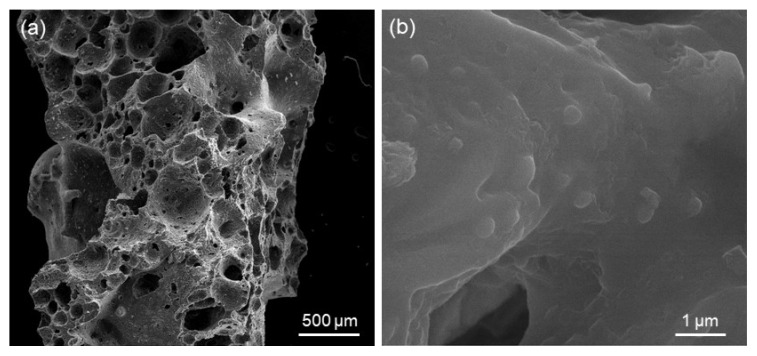
SEM analysis of 47.5B-derived scaffolds: (**a**) overview of the 3D pore-strut architecture (100×); (**b**) detail of the surface of scaffold struts (40,000×).

As widely demonstrated in previous literature, foam replication is a successful fabrication method to obtain porous ceramic and glass structures replicating the polymeric sacrificial template [19]. Figure 5a shows that porous glass-derived scaffolds with open, spheroidal pores were obtained (porosity above 70 vol.% and pore size above 100 µm, as recommended for bone tissue engineering scaffolds [20]); a comprehensive assessment of scaffold characteristics—including pore size distribution and permeability—was reported elsewhere [21]. Figure 5b displays the presence of crystals on the surface of scaffolds struts, which formed during sintering. As shown in previous studies on the same glass (47.5B compositional system) [22,23], devitrification took place at 750 °C, leading to the development of one crystalline phase, which was identified as combeite Na_2_Ca_2_(Si_3_O_9_). Interestingly, this newly-formed silicate phase was the same major phase crystallized in 45S5 Bioglass^®^-derived glass-ceramic products [24,25], which have been in clinical use since the 1990s [26]. Thus, no concerns were expected about the biocompatibility of 47.5B-based glass-ceramic scaffolds. Furthermore, early biological studies in vitro with mesenchymal stem cells and osteoblasts confirmed the good cytocompatibility of the 47.5B material [27].

**Figure 6:**
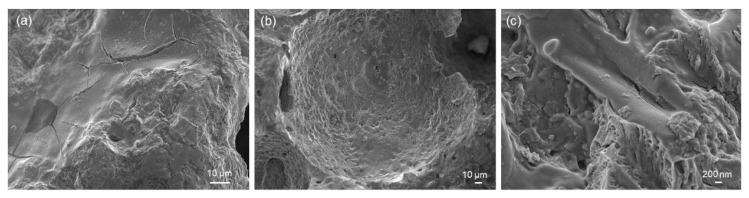
SEM analysis of HA-palmitate-coated scaffold: (**a**) coating on scaffold struts (3000×), (**b**) coating on the wall of a pore (1000×), and (**c**) detail of the coating (50,000×).

**Figure 7:**
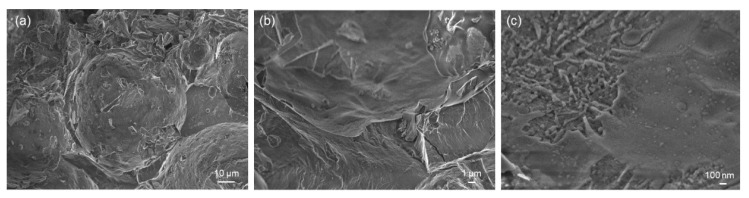
SEM analysis of HA-oleate-coated scaffold: (**a**) coating on scaffold struts and pore walls (1000×); (**b**,**c**) details of the coating (10,000× and 75,000×).

Figure 6 and Figure 7 show the results of HA-palmitate and HA-oleate coating deposition on glass-derived scaffolds. In both cases, the coating could be actually observed on the surface of struts as well as on the pore walls in the form of a “smooth skin”. The fibrous nature of the coating could also be appreciated at high magnification (Figure 6c and Figure 7c). Furthermore, the presence of the coating on both types of samples was confirmed by EDS analysis (above 30 wt.% of carbon was detected). Although the coating thickness, roughly ranging from a few tens to a few hundreds of nanometers, was not homogeneous on the scaffold surface, the immersion-based deposition procedure could be considered successful. Furthermore, the scaffold pores still remained open after the coating deposition, thus potentially allowing biological fluids to flow and living cells to spread in vivo. The decrease of porosity after both coating procedures is almost negligible (from 74 to 70 vol.%).

Glass-derived scaffolds coated with HA–fatty-acid conjugates were intended to be implanted in the body to promote bone tissue repair and regeneration. Therefore, investigating the persistence of the coating upon contact with aqueous solutions such as body fluids is key to determining the suitability of the material for the intended goal. In this regard, the decoating test provided early proof of the coating stability in the short term: in fact, for both types of samples, EDS analysis suggested that HA–fatty-acid conjugates were still present on the scaffold struts after the decoating procedure as the amounts of carbon before and after decoating were comparable (above 30 wt.% vs. 20–25 wt.%). A very high level of carbon could not be due to mere environmental effects.

During in vitro bioactivity tests, a moderate increase in the solution pH was observed (from 7.40 to 7.56 after 1 week for both sample types). This shift towards alkalinity was associated with the ion release from the surface of the scaffold to the simulated body fluid (SBF), which is in accordance with the bioactivity mechanism typical of SiO_2_-based bioactive glasses [28]. No problem related to the pH-related toxicity induced by the materials was expected as the pH peak reached upon immersion was around 7.56; such a slightly alkaline pH could even be beneficial to the viability and activity of bone cells, providing a stimulatory effect [29].

**Figure 8:**
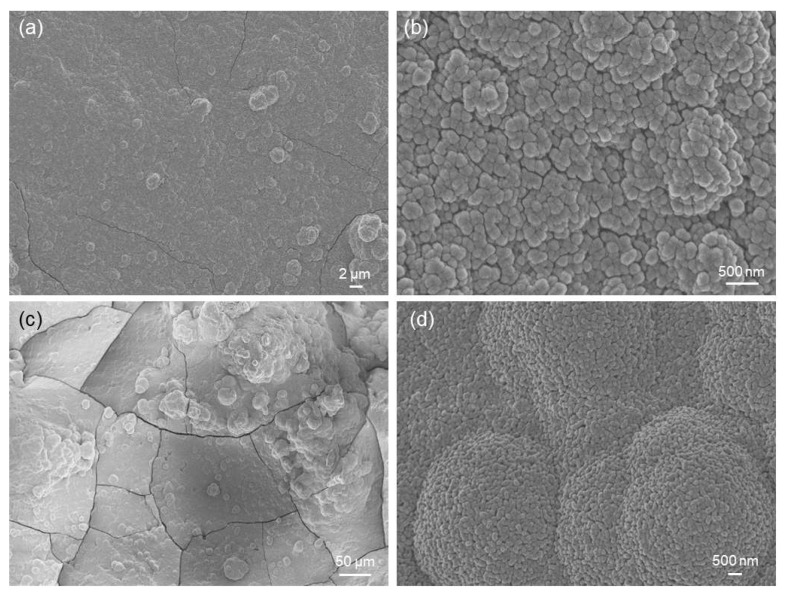
Results of in vitro bioactivity tests on HA-palmitate-coated scaffolds: SEM micrographs after (**a**,**b**) 48 h (5000× and 50,000×) and (**c**,**d**) 1 week of immersion in SBF (500× and 20,000×).

Figure 8a,b show the formation of a new phase on the surface of the scaffolds soaked for 48 h in SBF. This newly-formed phase is composed of calcium-phosphate (CaP) globular agglomerates, as compositionally revealed by EDS analysis, and this morphology (Figure 8b) is typical of hydroxyapatite-like layers formed on the surface of bioactive glasses after prolonged immersion in SBF.

**Figure 9:**
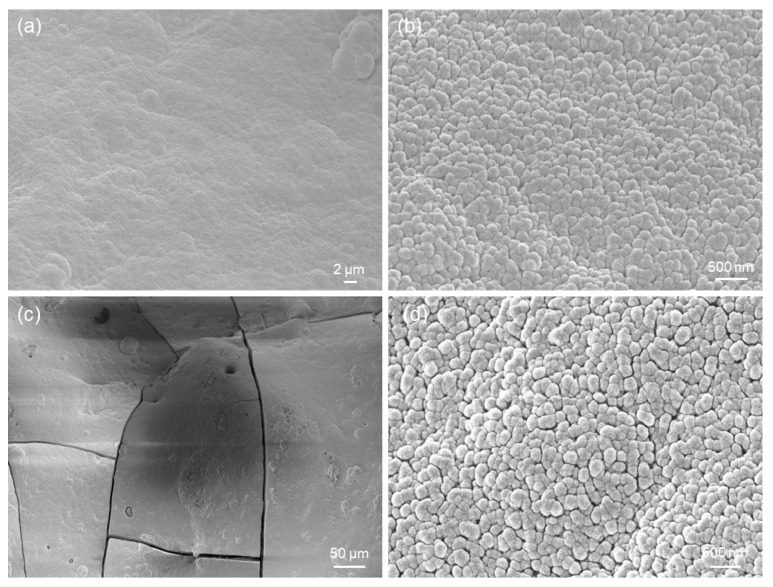
Results of in vitro bioactivity tests on HA-oleate-coated scaffolds: SEM micrographs after (**a**,**b**) 48 h (5000× and 50,000×) and (**c**,**d**) 1 week of immersion in SBF (500× and 50,000×).

The thickness of the CaP layer grew over time (Figure 8c), and the submicrometric structure of the agglomerates, which also assumed a typical “cauliflower-like morphology”, became well visible (Figure 8d). Cracking (Figure 8a,c) was the result of vacuum dehydration during sputter-coating prior to SEM analysis. After 7 days of immersion in SBF, EDS analysis revealed a Ca-to-P ratio of about 1.5 (atomic ratio), which is typical of the Ca-deficient hydroxyapatite formed on the surface of bioactive silicate glasses of various compositions [30,31,32,33]. A comparable Ca-to-P ratio was also found on the surface of robocast 47.5B scaffolds also soaked in SBF for 7 days. Similar results were found for HA-oleate-coated scaffolds, as displayed in Figure 9.

**Figure 10:**
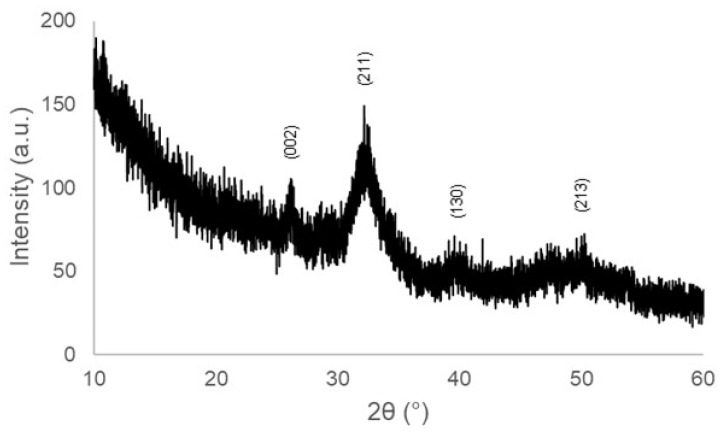
XRD on HA-palmitate-coated scaffolds after immersion for 1 week in SBF; the indexed peaks refer to hydroxyapatite.

The XRD pattern, displayed in Figure 10, revealed some reflections that can be attributable to hydroxyapatite (code 01-086-0740), in accordance with EDS results. The diffraction peaks are broad due to the submicrometric nature of hydroxyapatite, as shown in Figure 8d and Figure 9d.

Overall, the in vitro bioactivity results suggested that the presence of the HA–fatty acid conjugate coating had no detrimental impact on the apatite-forming ability of the scaffolds, which is preserved. In fact, the apatite-forming results shown here are analogous to those reported for uncoated scaffolds undergoing the same experimental conditions [22]. It is worth highlighting that the formation of a hydroxyapatite-like phase on the surface of biomaterials is key to promoting strong fixation with living tissues in vivo as osteoblasts preferably attach and proliferate on this nano-rough CaP layer, producing new bone [34]. These bioactive properties could be further potentiated by the presence of the coating; these aspects will deserve further investigation in vitro with cells and in vivo in future research.

## 3. Materials and Methods

### 3.1. Starting Reagents

The HA sodium salt from *Streptococcus Equi* (Mw = 15–30 kDa), fatty acids, and all solvents were purchased from Sigma–Aldrich (St. Louis, MO, USA).

Silicon oxide (SiO_2_), calcium carbonate (CaCO_3_), calcium phosphate (Ca_3_(PO_4_)_2_), magnesium carbonate hydroxide pentahydrate ((MgCO_3_)_4_Mg(OH)_2_·5H_2_O), sodium carbonate (Na_2_CO_3_), and potassium carbonate (K_2_CO_3_) were bought from Sigma-Aldrich and used as received for bioactive glass production.

### 3.2. Synthesis and Characterization of HA-Fatty Acids Conjugates

#### 3.2.1. Synthesis of HA–Fatty-Acid Conjugates

Fatty acid anhydrides were prepared as previously described [16]. In total, 10 mmol of the fatty acid was suspended in the appropriate volume of dichloromethane. Then, keeping the obtained mixture in an ice-water bath and under stirring, dicyclohexylcarbodiimide (5 mmol), which was previously solubilized in dichloromethane, was stepwise poured. The reaction was allowed for 2 h at 0 °C. Upon filtration, the solid N, N’-dicyclohexylurea was eliminated and the desired anhydride was isolated after solvent evaporation. Hyaluronan and the obtained fatty acid anhydride were mechanically milled in an agate mortar at the same weight ratio (1:1) by employing a catalytic amount (less than 1 mg) of base K_2_CO_3_. The reaction mixture was introduced in a 0.5–2 mL proper vial and exposed to microwaves for 2 min at 80 °C in a microwave oven (Initiator, Biotage Sweden AB, Uppsala, Sweden). Next, the mixture was solubilized in water and introduced in a 250 mL separatory funnel along with ethyl acetate to allow for the removal of the unreacted fatty acids and other by-products. After having neutralized the aqueous layer with aqueous 0.5 N HCl solution, a dialysis (membrane cut-off 6000–8000 Da) was performed for 1 day in Milli-Q water. The final product was recovered with a yield of 40%.

#### 3.2.2. FT-IR Characterization

The obtained HA conjugates underwent FT-IR spectroscopy using a FT/IR-4100 spectrometer (JASCO Corporation, Tokyo, Japan). Spectra were acquired by using the ATR accessory of the instrument (resolution: 4 cm^−1^, wavenumber range: 400–4000 cm^−1^). The typical bands related to the structure of the HA–fatty-acid conjugates were as follows: HA-oleate: 3428 cm^−1^ *ν*(O–H), 2923 cm^−1^ *ν*(C–H), 1737 cm^−1^ *ν*(C=O fatty acid ester), 1648 cm^−1^ *ν*_as_(COO^−^), 1413 cm^−1^ *ν*_as_(COO^−^), 1078 cm^−1^ *ν* and 1040 cm^−1^ *ν*(COC)_glycosidic bond ring_; HA-palmitate: 3415 cm^−1^ *ν*(O–H), 2919 cm^−1^ *ν*(C–H), 1740 cm^−1^ *ν*(C=O fatty acid ester), 1647 cm^−1^ *ν*_as_(COO^−^), 1415 cm^−1^ *ν*_as_(COO^−^), 1080 cm^−1^ *ν,* and 1047 cm^−1^ *ν* (COC)_glycosidic bond ring_.

### 3.3. Fabrication and Characterization of Glass-Derived Scaffolds

The glass used as a starting material for scaffold production was coded as “47.5B” and belonged to the oxide system 47.5 SiO_2_–2.5 P_2_O_5_–20 CaO–20 MgO–10 Na_2_O–10 K_2_O (mol. %). The 47.5B glass was synthesized by the melt-quenching route, as described elsewhere [22]. Briefly, powders of oxides and carbonates were first mixed in proper amounts (calculated from the nominal composition of the glass) on rotating rollers overnight, and then the blend was hand-pressed into a Pt crucible, covered with a Pt cap, and heated up to 1000 °C (heating rate: 12 °C/min, dwelling time: 10 min) into a high-temperature furnace (Nabertherm 1800 GmbH, Lilienthal, Germany) for achieving the decomposition of carbonates. After the removal of the Pt cap, the temperature was increased to 1500 °C (heating rate: 15 °C/min) and kept constant for 1 h to obtain a homogeneous melt. Finally, a “frit” was obtained after quenching the molten glass into distilled water, followed by ball milling and sifting below 32 µm.

Next, 47.5B-derived scaffolds were produced by soaking porous polyurethane templates in a glass-containing suspension (poly(vinyl alcohol): H_2_O: glass powder = 6/64/30, wt.%) according to a foam replication protocol described elsewhere [35]. After each immersion cycle, the sponge was placed onto a metallic grid and the excess suspension was squeezed out from the pores by applying an instantaneous compression load, as reported in [36]. The green bodies were dried overnight at room temperature and then heated to 750 °C for 3 h (heating rate: 5 °C/min) inside an electrical furnace (Nabertherm Muffle Furnace 1100°C L9/11/SKM/P330, Lilienthal, Germany) in order to sinter the inorganic particles and burn-off the sacrificial polymer.

The morphology of scaffolds was investigated by field-emission scanning electron microscopy (FE-SEM MERLIN, Carl Zeiss, Vienna, Austria). Prior to SEM analysis, an ultrathin Cr layer (7 nm) was sputtered on the surface of the samples to make them conductive. SEM analysis was performed using an accelerating voltage within 5–15 kV and was also carried out on the scaffolds after polymer-coating procedures and immersion studies in SBF.

The total porosity of scaffolds was estimated by density measurements through the assessment of mass-to-volume ratio.

### 3.4. Coating of HA–Fatty-Acid Conjugates on Bioactive Glass

Six milligrams (6.0 mg) of each HA–fatty acid sample was solubilized in milli-Q water. Then, the pH was adjusted by using an aqueous solution 0.5 N NaOH until the value of neutralization was reached. The bioactive glass scaffold was dunked in the obtained solution and left overnight at room temperature under gentle shaking. Then, the samples were extracted from the solution, lyophilized, and finally characterized by IR spectroscopy and SEM analysis.

The total porosity of coated scaffolds was estimated by density measurements.

### 3.5. Decoating of HA–Fatty-Acid Conjugates with Bioactive Glass

The bioactive glass scaffold coated with HA–fatty acid conjugate was dunked in milli-Q water and left at room temperature for 3–4 h under gentle shaking. Then, the sample was withdrawn from the solution and lyophilized to be finally characterized by SEM analysis.

### 3.6. In Vitro Bioactivity Test

These experiments, addressed to evaluate the apatite-forming ability of scaffolds, involved the immersion of samples in SBF. The testing solution was prepared following the procedure proposed by Kokubo and Takadama [36]. A mass-to-volume ratio of 1.5 mg/mL was used according to the recommendation of the Technical Committee 4 (TC04) in the International Commission on Glass (ICG) [37]. During the experiments, the HA–fatty acid conjugate-coated scaffolds were stored in sealed polyethylene bottles and placed inside a static incubator at 37 °C. After 48 h and one week, the samples were withdrawn from the container, gently washed with distilled water, left to dry overnight under a fume hood, and finally stored in a sealed plastic box prior to undergoing further analyses. The pH of the solution was recorded at each time point (triplicate samples).

The surface of the scaffolds was investigated by SEM; energy dispersive spectroscopy (EDS), enclosed in the equipment, was also carried out.

X-ray diffraction (XRD; X’Pert Pro PW3040/60 diffractometer, PANalytical, Eindhoven, Netherlands; Bragg–Brentano camera geometry with Cu Kα incident radiation λ = 0.15405 nm) was also performed on the intact scaffold after one week in SBF. The analysis parameters were 2θ range 10–70°, voltage 40 kV, filament current 30 mA, step counting time fixed to 1 s, and step size 0.02°. Phase identification was carried out by using X’Pert HighScore software 2.2b (PANalytical, Eindhoven, The Netherlands), relying on the PCPDFWIN database.

## 4. Conclusions

Foam-like silicate glass-ceramic scaffolds have been successfully coated with HA-palmitate and HA-oleate conjugates by a simple dipping procedure. The polymeric layer was rather uniformly distributed throughout the scaffold walls and struts, without occluding the 3D macroporous structure, as revealed by SEM analysis. The in vitro bioactivity of the HA–fatty-acid-coated scaffolds has been proved by the formation of globular nano-hydroxyapatite agglomerates after immersion for just 48 h in SBF. These novel multifunctional biomaterials show promise for bone repair due to the apatite-forming ability and tissue regenerative potential of the basic scaffold, combined with the well-known wound healing properties of hyaluronic acid and the antibacterial effect of natural fatty acids.

## Data Availability

Data are reported in the paper.
